# Cytokine preconditioning of engineered cartilage provides protection against interleukin-1 insult

**DOI:** 10.1186/s13075-015-0876-y

**Published:** 2015-12-14

**Authors:** Andrea R. Tan, Curtis D. VandenBerg, Mukundan Attur, Steven B. Abramson, Martin M Knight, J. Chloe Bulinski, Gerard A. Ateshian, James L Cook, Clark T. Hung

**Affiliations:** Department of Biomedical Engineering, Columbia University, 1210 Amsterdam Avenue, New York, NY USA; Department of Orthopedic Surgery, St. Luke’s-Roosevelt Hospital Center, 1000 10th Avenue, New York, NY USA; New York University Hospital for Joint Disease, 301 E. 17th Street, New York, NY USA; Institute of Bioengineering and School of Engineering and Materials Science, Queen Mary University of London, Mile End Road, London E1 4NS London, UK; Department of Biological Sciences, Columbia University, 1212 Amsterdam Avenue, New York, NY USA; Department of Mechanical Engineering, Columbia University, 500 W. 120th Street, New York, NY USA; Comparative Orthopaedic Laboratory, University of Missouri, 1100 Virginia Avenue, Columbia, MO USA

**Keywords:** Interleukin-1, Preconditioning, Cartilage tissue engineering

## Abstract

**Background:**

During osteoarthritis and following surgical procedures, the environment of the knee is rich in proinflammatory cytokines such as IL-1. Introduction of tissue-engineered cartilage constructs to a chemically harsh milieu may limit the functionality of the implanted tissue over long periods. A chemical preconditioning scheme (application of low doses of IL-1) was tested as a method to prepare developing engineered tissue to withstand exposure to a higher concentration of the cytokine, known to elicit proteolysis as well as rapid degeneration of cartilage.

**Methods:**

Using an established juvenile bovine model system, engineered cartilage was preconditioned with low doses of IL-1α (0.1 ng/mL, 0.5 ng/mL, and 1.0 ng/mL) for 7 days before exposure to an insult dose (10 ng/mL). The time frame over which this protection is afforded was investigated by altering the amount of time between preconditioning and insult as well as the time following insult. To explore a potential mechanism for this protection, one set of constructs was preconditioned with CoCl_2_, a chemical inducer of hypoxia, before exposure to the IL-1α insult. Finally, we examined the translation of this preconditioning method to extend to clinically relevant adult, passaged chondrocytes from a preclinical canine model.

**Results:**

Low doses of IL-1α (0.1 ng/mL and 0.5 ng/mL) protected against subsequent catabolic degradation by cytokine insult, preserving mechanical stiffness and biochemical composition. Regardless of amount of time between preconditioning scheme and insult, protection was afforded. In a similar manner, preconditioning with CoCl_2_ similarly allowed for mediation of catabolic damage by IL-1α. The effects of preconditioning on clinically relevant adult, passaged chondrocytes from a preclinical canine model followed the same trends with low-dose IL-1β offering variable protection against insult.

**Conclusions:**

Chemical preconditioning schemes have the ability to protect engineered cartilage constructs from IL-1-induced catabolic degradation, offering potential modalities for therapeutic treatments.

## Background

Articular cartilage is the connective tissue that lines the ends of diarthrodial joints and serves to bear load and provide lubrication during motion [1]. When the tissue is damaged through physical injury or disease such as osteoarthritis (OA), the healing response is limited due to its avascular nature and limited cellularity [2]. These types of injuries often lead to a disruption in the balance between anabolic and catabolic regulatory activities that lead to a phenotypic shift in chondrocytes, cell death, and an increase in expression of inflammation-related genes [3] that leads to a loss of cartilage matrix components and deterioration in the structural and functional properties of cartilage [4, 5]. Catabolic cytokines may also be generated by the fibroblast- and macrophage-like cells in the synovium, the thin layer that lines the non-articulating surfaces of diarthrodial joints and maintains a synovial fluid-filled cavity [6], in response to the breakdown products from damaged cartilage. Consequently, elevated levels of inflammatory cytokines such as interleukin-1 (IL-1) and tumor necrosis factor (TNF)-α have been measured in the synovial fluid during cartilage pathology [7, 8].

Though TNF-α is the dominant cytokine responsible for joint swelling and acute inflammation, it has been reported to be unlikely to play a role in tissue degradation [9]. In contrast, both forms of the IL-1 isoform, IL-1α and IL-1β, are reported to be responsible for cartilage erosion and sustained cell infiltration, though their concentrations are highly dependent on the stage of osteoarthritis [9] and the model system. As such, our lab and others have chosen to focus on the effect of IL-1 on culture systems to better understand the mechanisms behind erosive tissue breakdown and to potentially identify strategies for repair [10–15].

Strategies for using engineered constructs for cartilage tissue repair have focused on the implantation of tissues possessing the mechanical integrity and chemical fortitude to withstand the potentially harsh mechanochemical environment of the joint [10]. Catabolic mediators from inflammation [16] or the surgical intervention itself [17] have been shown to be especially pronounced in underdeveloped tissues [10, 18] in which cells are not fully embedded in the chondroprotective extracellular matrix (ECM) that accompanies construct maturity. Previous studies from our lab have reported that the lingering effects of IL-1 to immature constructs remains in long-term culture, even after the cytokine has been removed [10]. In order to successfully utilize these constructs for functional repair, it is important, therefore, to investigate potential strategies for preventing or mediating the effect of cytokines on the developing constructs.

In cardiovascular systems, cellular preconditioning schemes by mechanical and chemical manipulation have been used extensively to prime stem cells to a state in which they can withstand a harsh microenvironment [19–22]. In particular, brief exposure to a catabolic agent afforded longer-term protection against subsequent attacks, offering the most effective means of cytoprotection [23]. Previous studies have reported that hypoxia-inducible factor 1α (HIF-1α) serves as the predominant regulator of genes responsible for survival signaling and is activated when cells are exposed to a preconditioning scheme [19]*.* Motivated by the reported benefits of preconditioning, we hypothesized that the catabolic effects of injurious IL-1 on engineered cartilage could be decreased by initially preconditioning them with low doses of the cytokine.

This study investigates the effects of supplementing the culture media with a low concentration of IL-1. Specifically, the effects of a 1-week preconditioning treatment on developing engineered cartilage constructs is tested against exposure to a higher concentration of the cytokine, known to elicit proteolysis as well as rapid degeneration of cartilage [24]. This phenomenon is investigated first using a well-established juvenile bovine chondrocyte model [25], which has been shown to produce functional material properties (Young’s modulus (E_Y_)) and glycosaminoglycan (GAG) content similar to native tissue [26], providing a robust system to study potential therapies for osteoarthritis. Then, to provide additional support for the clinical feasibility of such a protection method, we subsequently translated this work to an adult canine model [27–29] to facilitate future in vivo studies in an established large preclinical animal model. This large animal model for articular cartilage repair is commonly used and ideal as the anatomy, physiology, and biomechanics of the patellofemoral joint is similar between species [30]. Additionally, canine models allow for multiple clinically relevant postoperative management strategies including non-weight-bearing slings, splints, external fixators with adjustable range of motion control, as well as underwater treadmills, leash walking, and athletic training activities. With both of these paradigms, the use of a physiologically relevant tissue to study this method of protection against cytokine exposure may shed light on the behavior of in situ cartilage during injury or disease.

## Methods

### Experimental design

**Fig. 1:**
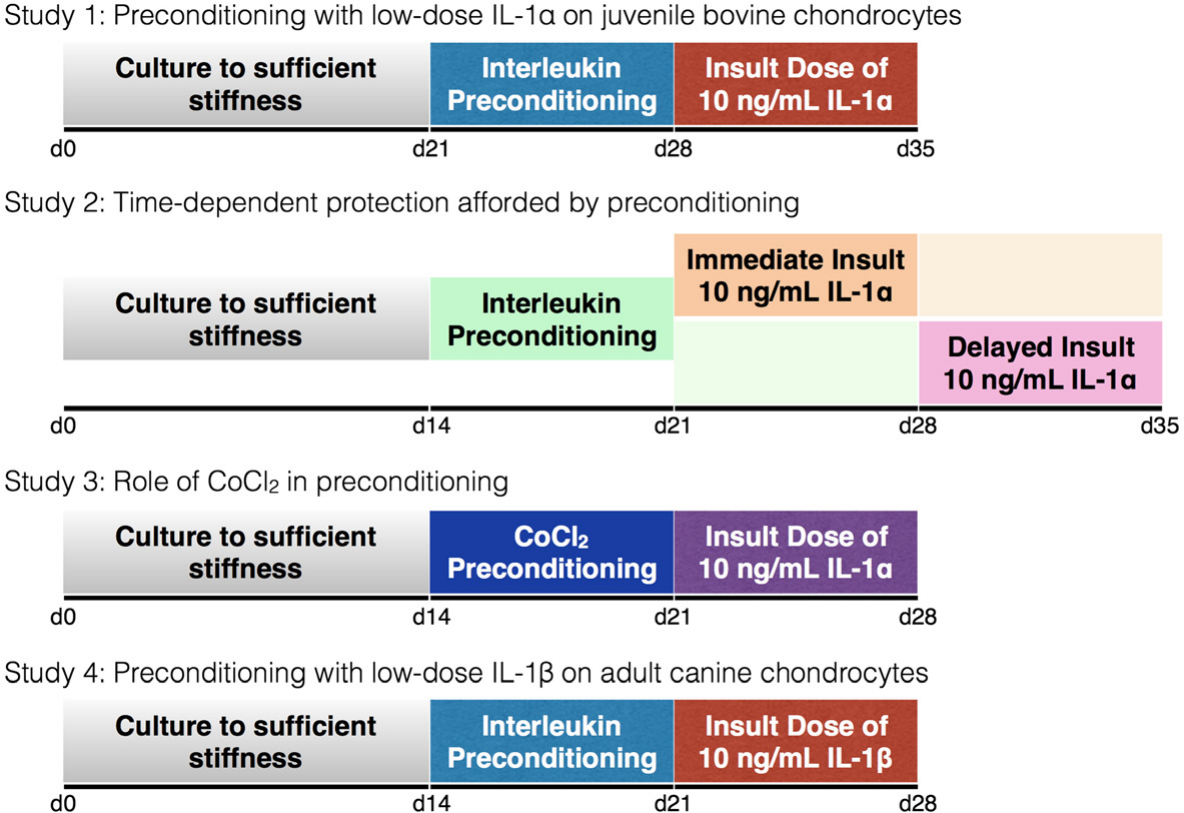
Schematic of experimental design. Studies 1 through 3 utilized the well-established juvenile bovine model system. Study 1 examined the protection afforded by low-dose IL-1α exposure. Study 2 investigated the time-dependent effects of preconditioning on protection by applying an immediate and delayed insult. Study 3 examined the effect of CoCl_2_ preconditioning to investigate a potential mechanism of protection. Study 4 explored the translational potential of preconditioning schemes by characterizing the effect on clinically relevant adult passaged canine chondrocytes. *CoCl*_*2*_ cobalt chloride, *IL-1* interleukin-1

Four consecutive studies are described here and laid out in Fig. 1. For the first three studies, we used a bovine model of chondrocytes from skeletally immature animals, as they are readily available, well characterized, and capable of growing robust tissue in culture (relative to adult cells). Additionally, use of juvenile bovine chondrocytes facilitates comparisons to our previous work, including the response of these cells to in vitro applications of IL-1α (10 ng/mL, [10]). In study 1, we investigated the protection afforded by low-dose preconditioning to understand how this chemical regime could be implemented for therapeutic applications. Briefly, constructs were allowed to elaborate a threshold amount of ECM (attaining a E_Y_ of at least 100 kPa), exposed to a low-dose concentration of IL-1α for 7 days and then subjected to an injurious level of cytokine for the final 7 days. In study 2, we looked further at the time-dependent profile of preconditioning on the protection afforded by varying the specific timing of the preconditioning scheme and the insult to answer two questions: (1) how long does protection last? and (2) how effective is the preconditioning scheme after an interim period? Then, in study 3, we investigated the potential role of the survival signal hypoxia-inducible factor (HIF)-1α in affording this protection, similar to the mechanism suggested for hypoxic and ischemic preconditioning schemes [31, 32]. For this purpose, juvenile bovine constructs were preconditioned with cobalt chloride (CoCl_2_), a chemical inducer of HIF-1α, and then exposed to an insult dose of IL-1α to investigate whether HIF-1α upregulation was sufficient to provide protection against catabolic effects. Finally, in study 4, we investigated whether the noted trends carry over to a preclinical canine model using chondrocytes from skeletally mature animals.

### Tissue isolation and cell culture

All bovine studies were Columbia Institutional Animal Care and Use Committee (IACUC)-exempt. Canine studies were performed under University of Missouri IACUC approval (ACUC number 7332). Articular cartilage was harvested from knee joints of either freshly slaughtered 2–4-week-old bovine calves or from adult dogs. Four to six joints were used for each experiment and cells were pooled from all joints. In study 4, cartilage was digested in high-glucose Dulbecco’s modified Eagle’s medium (DMEM) with collagenase IV (Worthington Biochemical Corporation, Lakewood, NJ, USA) for 11 h at 37 °C with shaking. Cell suspensions were filtered through a 70-μm porous mesh and sedimented in a bench-top centrifuge for 15 min at 1500 g. Viable cells were counted with a hemocytometer and trypan blue and plated at high density (20 × 10^3^ cells/cm^2^) in a growth factor cocktail (1 ng/mL transforming growth factor (TGF)-β1, 5 ng/mL basic fibroblast growth factor (bFGF), and 10 ng/mL platelet-derived growth factor (PDGF)-ββ [27, 33]). At confluence, cells were trypsinized, resuspended (60 × 10^6^ cells/mL), and mixed in equal parts with 4 % low-gelling agarose (type VII, Sigma-Aldrich, St. Louis, MO, USA) at 37 °C. The chondrocyte/agarose mixture was cast into slabs and cores were produced using a sterile, disposable biopsy punch (Integra Miltex, York, PA, USA) to yield final dimensions (4 mm × 2.34 mm thick). Constructs were cultured in DMEM supplemented with 1× penicillin, streptomycin, fungizone (PSF, Sigma-Aldrich), 0.1 μM dexamethasone, 40 μg/mL L-proline, 100 μg/mL sodium pyruvate, and 1× ITS+ premix (insulin, human transferrin, and selenous acid, Becton Dickinson, Franklin Lakes, NJ, USA). Medium was further supplemented with 50 μg/mL ascorbate 2-phosphate daily and 10 ng/mL TGF-β3 (Invitrogen, Carlsbad, CA, USA) for the first 14 days of culture and was changed every other day.

### Preconditioning and insult

Earlier studies have identified that constructs must comprise sufficient extracellular matrix (ECM) to withstand the harsh catabolic effects of IL-1 [10]. Accordingly, for the current studies, constructs were allowed to attain an equilibrium Young’s modulus (E_Y_) of at least approximately 100 kPa (for bovine) and approximately 150 kPa (for canine) before the application of any cytokine. For study 1, IL-1α, the more potent of the two isoforms in juvenile bovine agarose-chondrocyte constructs [11], was administered (0.1 ng/mL, 0.5 ng/mL, 1.0 ng/mL) for 7 days before an insult dose (10 ng/mL) was added to the culture medium for the final 7 days. In study 2, a subset of the preconditioning doses from study 1 was administered for 7 days based on the success at mitigating the effects of interleukin exposure (0.1 ng/mL, 0.5 ng/mL) when constructs reached sufficient ECM deposition. The 7-day insult dose was then applied either immediately following the preconditioning (immediate insult, Fig. 1, orange block) or after 7 days had elapsed (delayed insult, Fig. 1, pink block). In study 3, in place of preconditioning with interleukin dosing, constructs were exposed to media supplemented with 100 μM CoCl_2_ before the insult dose was administered. To parallel the conditions of study 1, in study 4, IL-1β (0.2 ng/mL, 1.0 ng/mL, 2.0 ng/mL), the more potent isoform for canine constructs, was used [28, 29] for 7 days before an insult dose (10 ng/mL) was added to the culture medium for the final 7 days. For all studies, a negative control group was included where samples were handled similarly but no preconditioning or insult dosage was applied. A positive control group (labeled 0 ng/mL) was also included where preconditioning was not applied, but samples were exposed to the insult dose of IL-1. During the period of cytokine (both preconditioning and insult) and CoCl_2_ exposure, all media was void of dexamethasone, previously shown to counter the catabolic effects of IL-1 [10].

### Mechanical testing

Constructs were tested for their equilibrium E_Y_ and dynamic modulus (G*) in unconfined compression using a custom computer-controlled system [34]. An initial 0.02 N tare load was applied, followed by compression to 10 % strain, at a strain rate of 0.05 % s^−1^. After stress relaxation was achieved, a 2 % peak-to-peak strain was superimposed at 0.01Hz. E_Y_ was measured from the stress–relaxation equilibrium response and is used as a measure of the equilibrium behavior of cartilage after an applied load has been applied. G*, considered to be a more physiologically relevant measurement of cartilage behavior in response to applied cyclic loads, was determined from the slope of the stress–strain response under dynamic loading.

### Biochemical analysis

After material testing, half of the construct was dried and digested in proteinase K solution overnight at 56 °C, as described previously [35], and the other half was preserved for histology (see below). Specifically, the biochemical content of each sample was assessed by measuring the sample wet weight, lyophilizing, and then measuring the dry weight. Construct swelling was quantified by measuring the gross water content of the constructs [26]. Following digestion, one aliquot was analyzed for GAG content via the 1,9 dimethylmethylene blue dye-binding assay. A second aliquot was hydrolyzed with 12 N HCl at 110 °C for 16 h, dried, and resuspended in assay buffer. Orthohydroxyproline (OHP) content was determined using a colorimetric assay via a reaction with chloramine T and dimethylaminobenzaldehyde, scaled for microplates [35]. Overall collagen content was calculated using a 1:7.64 OHP-to-collagen mass ratio [36]. Total double-stranded DNA content was assessed by the Picogreen assay, following the manufacturer’s standard protocols.

### Histological analysis

The other half of each sample was fixed in acid-formalin, paraffin-embedded, sectioned (8 μm thick), and stained for histology to assess cellular (hematoxylin and eosin), proteoglycan (Safranin O), and collagen (Picrosirius Red) distribution and organization. Immunohistochemistry was also performed to confirm the development of collagen II in constructs. Briefly, tissue sections were digested in 5.0 mg/mL testicular hyaluronidase (Sigma-Aldrich), swollen in 0.5 mol/L acetic acid, and blocked in 10 % normal goat serum (NGS). Sections were labeled with types I and II collagen (Abcam, Cambridge, MA, USA) and visualized with Alexa Fluor 488-conjugated secondary antibody labeling (Molecular Probes, Eugene, OR, USA) and diamidino-2-phenylindole nuclear counterstaining (Molecular Probes). Sections were observed on a laser scan confocal microscope and a tile scan was performed to visualize the entire tissue slice (Carl Zeiss Microscopy, LLC, Thornwood, NY, USA).

### Statistics

Statistical analyses were performed using two-way analysis of variance (ANOVA) with Tukey’s honest significant difference post hoc tests (Statistica; StatSoft Inc., Tulsa, OK, USA), with α = 0.05 and statistical significance set at *p* ≤ 0.05 to compare groups across day and treatment. Data is reported as the mean and standard deviation of four to five samples per time point and group.

## Results

### Study 1: low-dose preconditioning affords protection against subsequent IL-1 exposure

**Fig. 2:**
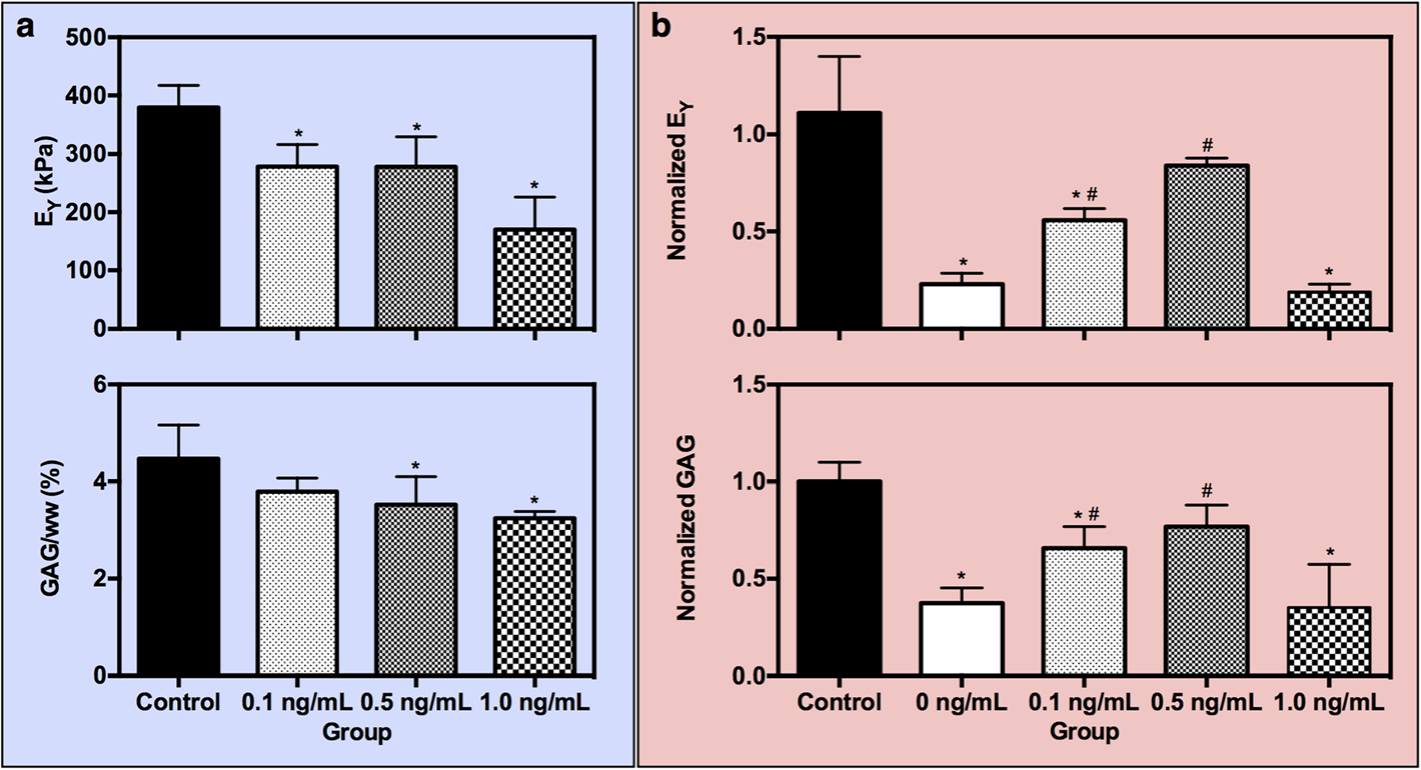
Study 1. (**a**) Young’s modulus (E_Y_) and GAG content at day 28 as a result of preconditioning juvenile bovine constructs with low doses of IL-la. (**b**) Normalized E_Y_ and GAG content at day 35 after 7 days of exposure to an insult dose of IL-lα (10 ng/mL). ^*^ *p* < 0.05 vs. control, ^#^ *p* < 0.05 vs. positive control (0 ng/mL). *GAG* glycosaminoglycan, *IL-1* interleukin-1

All four studies were started on constructs that had matured, i.e., elaborated sufficient ECM to resemble in situ cartilage. For these constructs, maturation in culture to day 21 yielded significantly greater mechanical stiffness and biochemical content compared with initial day 0 conditions (E_Y_ = 96.8 ± 22.3 kPa, GAG = 1.16 ± 0.26 %/ww). Constructs were then exposed to low doses of IL-1α. At day 28, control constructs continued to elaborate matrix and increase stiffness, while those constructs exposed to low doses of IL-1α showed significantly lower mechanical stiffness and GAG composition, demonstrating a concentration-dependent degrading action of the cytokine. For example, with 0.1 ng/mL and 0.5 ng/mL doses, slight tissue degradation was noticeable, as evidenced by the 25 % drop in mechanical properties compared to control samples (*p* < 0.05, Fig. 2a). A corresponding 15 % and 22 % drop in GAG content, respectively, was noted for those same low-dose concentrations, however the 0.5 ng/mL preconditioning dose elicited a significantly lower GAG content relative to control samples (*p* = 0.03, Fig. 2a). When 1.0 ng/mL IL-1α was introduced into the culture medium, however, the catabolic effects were much more severe, significantly lowering mechanical stiffness by nearly 55 % (*p* = 0.0001). Interestingly, the decrease in GAG content did not mirror the significant drop in mechanical properties; instead, a more moderate 27 % decrease was measured compared to control samples (*p* = 0.004). Collagen content was unchanged across all groups.

Following 7 days of insult culture (10 ng/mL IL-1α), a differential response was noted between positive control and preconditioned constructs. To evaluate the effect of preconditioning, construct properties were normalized to negative control values. Notably, positive control samples showed a significant degree of degradation, retaining only 23 % of the construct’s mechanical stiffness (*p* < 0.0001) and 40 % of the construct’s GAG content (*p* < 0.0001, Fig. 2b). In comparison, preconditioning constructs with low doses of IL-1α the week prior to the insult culture afforded dose-dependent protection against catabolic degradation. With 0.1 ng/mL IL-1α preconditioning, slight protection was afforded. That is, though significantly lower in mechanical stiffness (*p* = 0.001) and GAG content (*p* = 0.007) than negative control samples, these constructs exhibited significantly greater mechanical stiffness (*p* = 0.04) and GAG content (*p* = 0.04) than positive control samples. In contrast, 0.5 ng/mL IL-1α preconditioning provided even greater protection against degradation, with mechanical stiffness and GAG content higher than positive control samples (*p* < 0.05), and more importantly, similar to negative control constructs (*p* > 0.05, Fig. 2b). There is a limit to the observed protection afforded by preconditioning; when constructs were exposed to the highest preconditioning dose of IL-1α (1.0 ng/mL), they showed a similar degradation profile as that seen with positive control samples, exhibiting comparable decreased mechanical stiffness (*p* = 0.99) or GAG content (*p* > 0.99). Collagen content was maintained across all groups, regardless of IL-1 exposure. Similarly, DNA content and construct swelling was not significantly altered across any group for all time points (data not shown, *p* > 0.05).

### Study 2: protection against IL-1 effects is sustained after delayed insult

Preconditioning schemes were capable of maintaining the mechanical integrity and biochemical makeup of constructs even over extended timescales. During the preconditioning phase (Fig. a, b green shaded region), low levels of IL-1α (0.1 ng/mL and 0.5 ng/mL) exposure did not significantly alter the mechanical or biochemical composition of the constructs relative to control samples (*p* > 0.05). Similarly, DNA content and construct swelling was not significantly altered across any group (data not shown, *p* > 0.05).

**Fig. 3:**
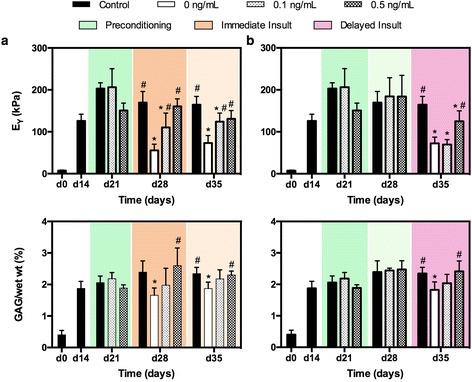
Study 2. Young’s modulus (E_Y_) and GAG (%/ww) for juvenile bovine constructs over time for (**a**) immediate and (**b**) delayed insult. Color blocks indicate treatment group (Fig. 1). ^*^ *p* < 0.05 vs. control, ^#^ *p* < 0.05 vs. positive control (0 ng/mL). *GAG* glycosaminoglycan

The protection afforded by preconditioning following immediate insult (Fig. 3a, dark orange shaded region) mimicked the findings from study 1: a significant drop in properties was observed for positive control samples (*p* < 0.0001). These catabolic changes were mitigated with 0.1 ng/mL preconditioning exposure (*p* = 0.01) and protected against with 0.5 ng/mL preconditioning exposure (*p* = 0.93). When insult was removed for the following 7 days, similar trends were noted and the mechanical properties and biochemical content were sustained (Fig. 3a, light orange shaded region).

When samples were cultured for a 7-day interim period following preconditioning, mechanical properties and biomechemical content were unchanged (Fig. 3b, light green shaded region). Delayed insult (Fig. 3b, pink shaded region) produced similar effects on positive control samples as after immediate insult (% degradation_immediate_ = 0.67 ± 0.09 vs. %degradation_delayed_ = 0.56 ± 0.09, *p* = 0.13). Preconditioning with 0.1 ng/mL IL-1α, however, did not show the same protective benefits seen previously; instead, constructs exhibited a significant decrease in Young’s modulus (*p* < 0.0001), similar to positive control constructs (*p* = 0.62). Biochemical content was unchanged from control samples (*p* > 0.05). In contrast, constructs preconditioned with 0.5 ng/mL IL-1α continued to exhibit partial protection from catabolic degradation, retaining 76 % of their mechanical stiffness and comparable GAG content (*p* = 0.98) compared to control samples. This protection reflects a significant improvement in mechanical functionality compared to positive control samples (*p* = 0.005).

**Fig. 4:**
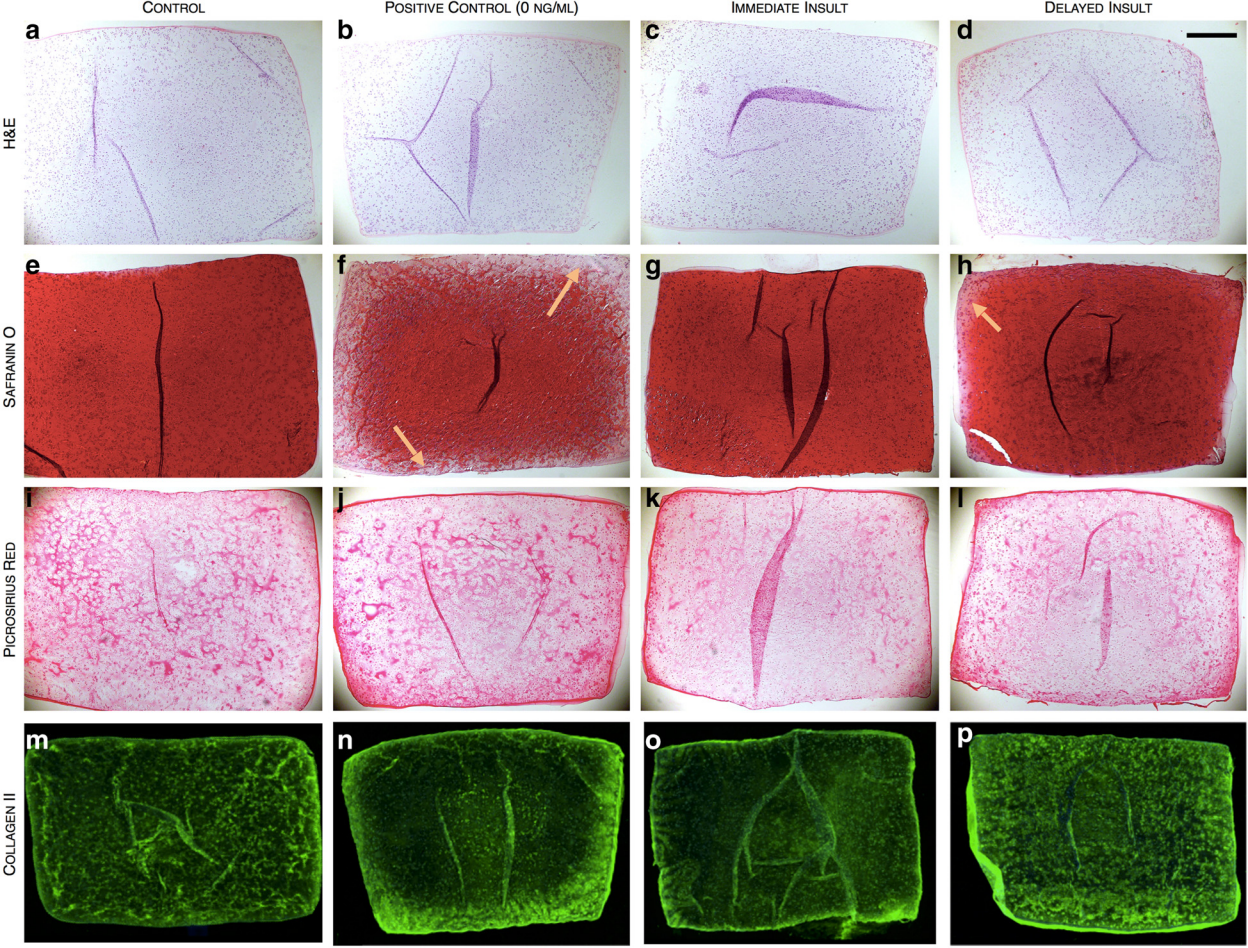
Study 2. Histological stains for cellularity (H&E), GAG (Safranin 0), bulk collagen (Picrosirius Red), and collagen II (Alexa Fluor 488) distribution for juvenile bovine constructs exposed to IL-1α insult in the (**b**, **f**, **j**, **n**) absence of or after (**c**, **g**, **k**, **o**) immediate and (**d**, **h**, **i**, **p**) delayed insult schemes (0.5 ng/mL)*.* Scale bar = 0.5 mm. Arrows indicate depleted ECM matrix. *ECM* extracellular matrix, *GAG* glycosaminoglycan, *H&E* hematoxylin and eosin, *IL-1* interleukin-1

For both immediate and delayed insult schemes, 0.5 ng/mL IL-1α offered a degree of protection against the supraphysiologic IL-1α exposure; histological stains confirmed these findings. Control constructs exhibited uniform cellularity (Fig. 4a) throughout the construct that produced robust GAG staining (Fig. 4e) accompanied by pockets of intense collagen formation throughout the construct (Fig. 4i). Immunohistochemistry confirmed the presence of collagen II, with patterns of greater intensity mimicking the pockets identified with Picrosirius Red stain (Fig. 4m). Positive control samples revealed a large distinct border of GAG depletion (Fig. 4f, denoted by arrows), though overall bulk collagen distribution was unchanged from control samples (Fig. 4j vs. 4i). Specific collagen II staining of positive control samples however, revealed an intense counter border to the depleted GAG molecules (Fig. 4n). Constructs exposed to 10 ng/mL IL-1α immediately following preconditioning revealed a similar appearance as control constructs (Fig. 4g, k, o). In contrast, when constructs were exposed to delayed 10 ng/mL IL-1α insult, although overall GAG content was statistically similar, distribution was partially heterogeneous across the entirety of the construct with a slight depletion of GAG at the border of the construct (Fig. 4h, yellow arrow).

### Study 3: CoCl_2_ preconditioning offers similar protection scheme

**Fig. 5:**
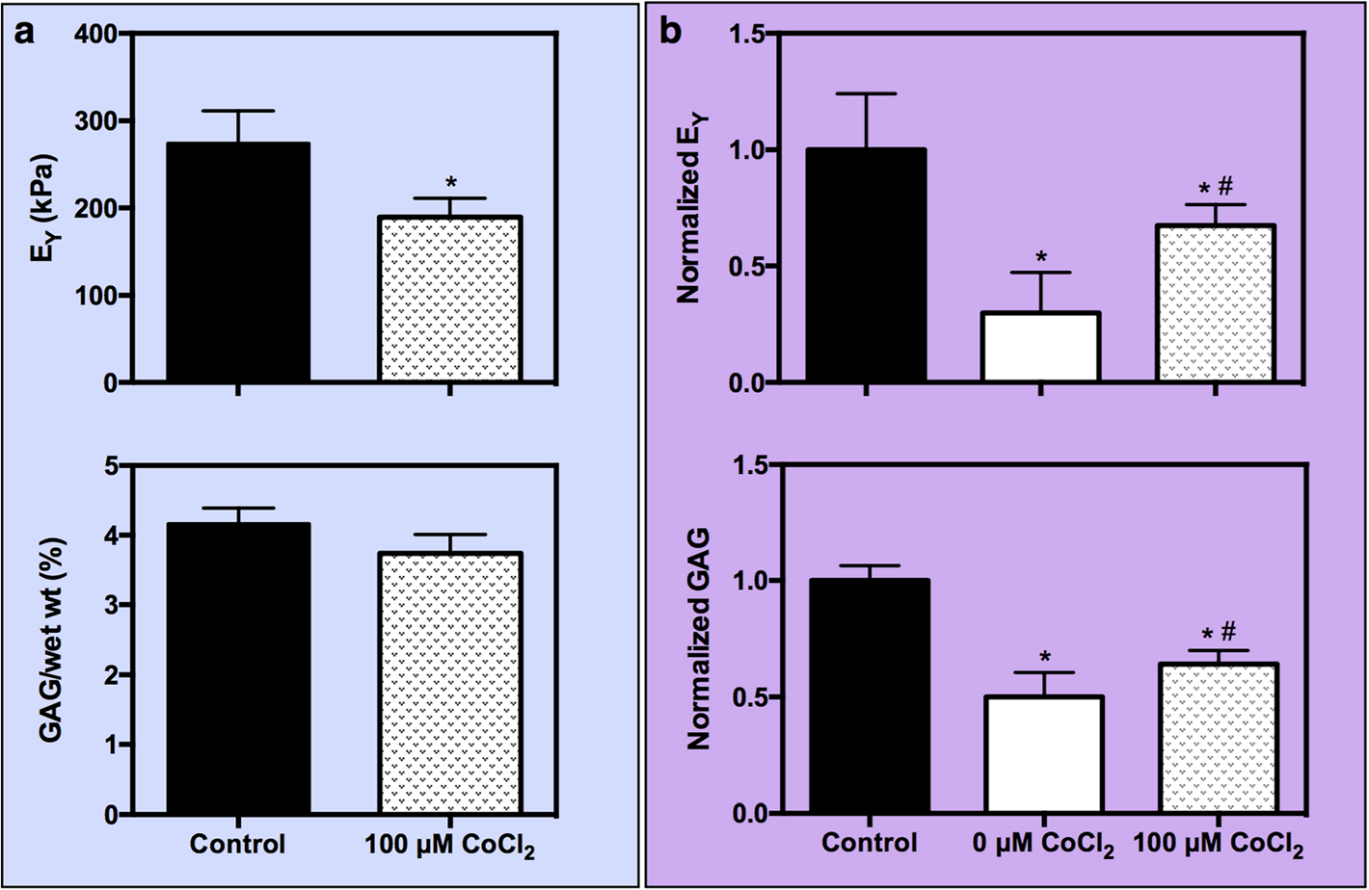
Study 3. (**a**) Young’s modulus (E_Y_) and GAG content at day 21 after 7 days of reconditioning juvenile bovine chondrocytes with 100 μM CoCl_2_. (**b**) Normalized E_Y_ and GAG content at day 28 after insult dose of 10 ng/mL IL-lα was administered. ^*^ *p* < 0.05 vs. control, ^#^ *p* < 0.05 vs. positive control (0 μM CoCI_2_). *GAG* glycosaminoglycan, *IL-1* interleukin-1

In study 3, in lieu of preconditioning with low doses of cytokine, we explored the use of CoCl_2_ as a means of activating HIF-1α transcription. On day 14, prior to the start of CoCl_2_ preconditioning, constructs possessed appreciable ECM deposition and associated mechanical properties similar to those in mature constructs used in studies 1 and 2 (E_Y_ = 174 ± 13.8 kPa; GAG = 3.21 ± 0.20 %/ww). Following preconditioning with CoCl_2_, constructs exhibited significantly lower stiffness (*p* = 0.0076, Fig. 5a), but comparable dynamic modulus, GAG, and collagen content compared to control constructs (*p* > 0.05). Following insult with 10 ng/mL IL-1α, positive control samples behaved similarly to those in studies 1 and 2, significantly degrading in mechanical properties (*p* < 0.0001) and GAG content (*p* < 0.0001) compared to control samples. Collagen content for these positive control samples was statistically similar to control samples. CoCl_2_-preconditioned constructs showed resistance to the catabolic degradation imparted by IL-1α and were capable of retaining more of their GAG content (*p* = 0.04, Fig. 5b), leading to improved mechanical properties (*p* = 0.02) compared to positive control samples. Contrary to previous findings that collagen content is unchanged following in vitro IL-1α application, here, we note a decrease in collagen content for CoCl_2_-preconditioned constructs (*p* < 0.0001), though DNA content and construct swelling was not significantly altered across any group (data not shown, *p* > 0.05).

### Study 4: exploring clinical translation feasibility with adult canine chondrocytes

**Fig. 6:**
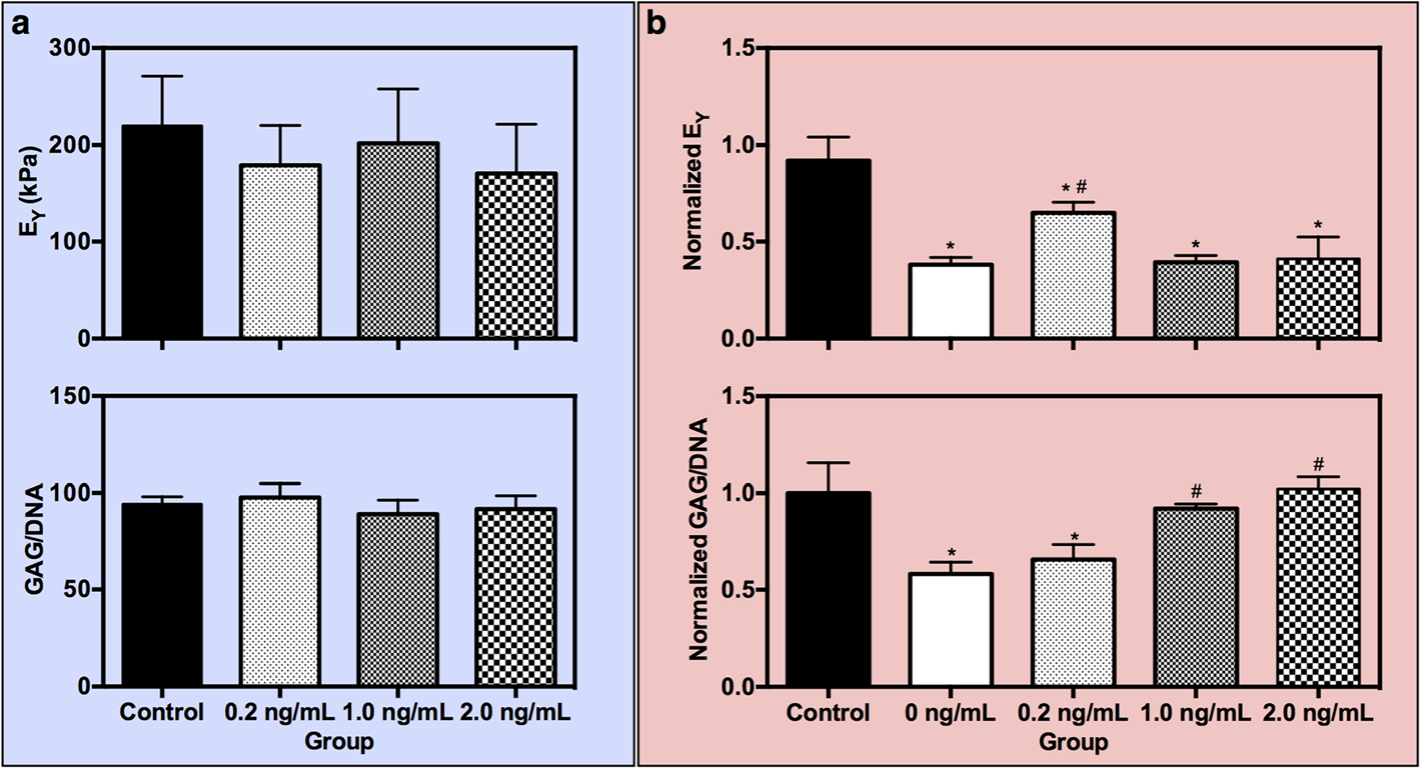
Study 4. (**a**) Young’s modulus (E_Y_) and GAG content at day 21 as a result of preconditioning adult canine chondrocytes with low doses of lL-1β. (**b**) Normalized E_Y_ and GAG content at day 28 after 7 days of exposure to an insult dose of IL-lα (10 ng/mL). ^*^ *p* < 0.05 vs. control, ^#^ *p* < 0.05 vs. positive control (0 ng/mL). *GAG* glycosaminoglycan, *IL-1* interleukin-1

Similar to bovine chondrocytes, adult canine chondrocytes first elaborated significant amount of ECM resulting in increased mechanical properties and biochemical composition by day 14 (E_Y_ = 191 ± 17.0 kPa, GAG = 3.33 ± 0.16 %/ww). By day 21, after 7 days of low-dose concentrations of IL-1β, all constructs showed continued mechanical functionality that was not significantly altered by the addition of the cytokine (*p* > 0.05, Fig. 6a). However, unlike for bovine chondrocytes, constructs comprised of canine chondrocytes revealed a change in DNA content following IL-1β application. To account for this change, GAG content was reported as a function of DNA content, reflecting the biosynthetic output of the cells. Accordingly, the GAG production after low-dose IL-1β preconditioning was similar to control samples (*p* > 0.05, Fig. 6a). Collagen content and construct swelling were also unchanged.

Following cytokine insult (10 ng/mL IL-1β), positive control samples exhibited significant decreases in mechanical stiffness (*p* < 0.0001, Fig. 6b) and GAG content (*p* < 0.0001, Fig. 6b). Low-dose concentrations of 0.2 ng/mL IL-1β offered mediation of catabolic degradation reflected in mechanical properties by retaining a significantly higher Young’s modulus compared to positive control samples (*p* = 0.01, Fig. 6b). The partial protection noted for mechanical properties was not reflected in the biochemical composition of the samples, however; GAG/DNA produced by cells exposed to 0.2 ng/mL IL-1β was statistically similar to positive control samples (*p* = 0.74). The reverse trend was seen for higher (1.0 and 2.0 ng/mL) preconditioning doses of IL-1β; mechanical properties were similarly affected by the insult level of cytokine as positive controls (*p* > 0.05, Fig. 6b) though GAG content recovered and was comparable to negative control samples (*p* < 0.05). Collagen content and construct swelling were unaffected for all groups.

## Discussion

Cartilage grafts introduced to an injured or arthritic joint are likely to be subjected to a cytokine-rich environment. Thus, in order to promote engineered cartilage as a clinically relevant replacement tissue, the construct must be able to survive the chemical and mechanical loads that are imparted upon implantation. In this context, we explored a protection method by which functional engineered cartilage constructs that are capable of attaining native functional properties (approximately 400–1000 kPa [26, 37]; canine: approximately 190–380 kPa [27, 38]) can better withstand the harsh chemical environment of the joint. To our knowledge, this is the first study to show that chemical preconditioning schemes impart protection against injurious IL-1 exposure. Specifically, by varying the preconditioning dose, we were able to identify conditions under which mechanical properties and biochemical composition were significantly retained, despite subsequent exposure to injurious levels of the cytokine, even when the insult is delayed. These results support our hypothesis regarding the benefits of cytokine preconditioning and suggest that preconditioning in a clinical therapeutic context may be feasible as time between preconditioning and insult does not completely remove the protective benefits observed. Furthermore, we have noted successful translation of these results from a bovine model into a large preclinical animal model system. Future work exploring the mechanisms behind this protection and the differential mediators is necessary; nevertheless, this early data serve as encouragement for the eventual application of preconditioning techniques to therapies for cartilage repair in the clinic.

The use of clinically available human cells in functional tissue engineering may introduce additional factors that may influence the utility of preconditioning as we have described here. For example, while differentiated adult chondrocytes may be obtained from a patient’s own healthy, non-load-bearing cartilage, this may lead to donor site morbidity and further tissue degeneration [39]. As an alternative, chondrocytes from the diseased knee may also be harvested during preliminary debridement procedures. It is unknown, however, how such cells isolated from diseased (e.g., arthritic) tissue will function. Further assessment is needed to understand whether the cells maintain an osteoarthritic phenotype and whether the cell memory of the in vivo environment will influence the preconditioning scheme. To circumvent these potential problems, it may be more advantageous to use chondrocytes derived from juvenile articular cartilage, as they have already been shown to rapidly grow and produce significantly greater levels of cartilage macromolecules than cells derived from adult cartilage [40].

While many studies employ a classical concept of preconditioning involving cyclical exposure to brief episodes [20, 41, 42], other studies utilized a single long-term exposure [21, 31, 43, 44] to initiate survival signaling that was then followed by the harmful insult. We chose to adopt the latter as the longer preconditioning time frame would increase the likelihood of the cytokine diffusing fully through the agarose construct within the preconditioning period. It is important to note, however, that while the protective effect we observed here has been previously noted with other chemical schemes to prepare individual cells against harsh conditions [19, 23], this is the first time the effect has been observed for complex whole tissues that include elaborated extracellular matrix. The dense matrix in cartilage constructs confounds attempts to understand the potential mechanism(s) of protection. That is, the mode of protection may arise from an altered state of the cells themselves, or because the preconditioning has induced the cells to develop a chondroprotective matrix that is able to mediate the effects of the cytokines.

Previous studies have supported the idea that a cell’s tolerance to subsequent insult events is afforded by activation of a survival signal that depends on multiple signaling pathways or on the release of chemical activators such as adenosine or nitric oxide [41]. Furthermore, other studies have found that modulation of growth factor expression either through the use of specific factors during expansion [23] or through genetic manipulation can produce similar beneficial preconditioning effects. Further studies are necessary to systematically identify which pathways are involved in the protective process; however, we can postulate that, in agreement with previous preconditioning studies [31, 32] and as supported by the results we observed, the HIF-1α pathway has a central role in the activation of this survival signal against IL-1 catabolic degradation. Indeed, other groups have found the transcription factor regulates not only proinflammatory mediators, but also glucose transport, anaerobic energy generation, and matrix synthesis. [45, 46]. These downstream effects may concomitantly work to preserve cartilage integrity during insult.

Despite the protection afforded by preconditioning, however, we noted a decrease in collagen content after preconditioning with CoCl_2_ (data not shown) that was not seen with IL-1 preconditioning groups. This may point to the possibility that a downstream effect of inducing hypoxia may be an upregulation of collagenases that did not occur in our other preconditioning scheme. Additionally, though we did not directly assay for HIF-1α upregulation, previous studies have found an association between hypoxia induction with the upstream activation of the p38 mitogen-activated protein (MAP) kinase pathway [47]. p38 MAP kinase has been found to upregulate cytokine production via direct phosphorylation of transcription factors [48], possibly leading to additional catabolic effects on the bulk construct. Further work will be necessary to fully elucidate this possibility. Nevertheless, preconditioning with a low dose of IL-1 had a similar effect as preconditioning with induced hypoxia, suggesting that the downstream effect of IL-1 preconditioning may be attributable to upregulation of HIF-1α or a similar effect on cellular transcription.

The heightened generation of a chondroprotective matrix is unlikely to be the only cause of the protection. Previous studies have suggested that pools of GAG in the pericellular region and interterritorial regions change with culture time such that changes to the local environment of the cell alter the cell’s function and response to external stimuli such as IL-1 [49–52]. This local matrix forms around day 4 in culture, making the cells stiff inclusions in a soft matrix (agarose gel), but with time in culture, this cell-associated matrix modulates the sensitivity to chemical factors. However, under our current experimental conditions, unlike previously reported studies [10], we have observed vulnerability to IL-1 regardless of mechanical integrity or biochemical composition, thus ruling out extensive cytoprotection by matrix components. Furthermore, samples with the same composition and mechanical integrity responded differently to insult levels of IL-1 depending on their preconditioning (none vs. low-dose IL-1 vs. CoCl_2_ treatment). It is important to note, however, that our measurements of bulk properties and biochemical constituent content may not detect compositional changes to the matrix. Changes in the composition of matrix could be responsible for the protection against IL-1 that preconditioning with either low-dose IL-1 or CoCl_2_ elicits. Further analysis, however, using comprehensive proteomics gene expression arrays will be required to better understand whether changes to the matrix or subsequent cellular changes are responsible for resisting the catabolic damage brought about by IL-1 exposure.

Finally, although the concentration of the cytokine used to impart damage to the tissue was on par with concentrations typically utilized for in vitro studies (5–20 ng/mL [14, 53, 54]), the dose we used is well above doses found in vivo (0.01–0.2 ng/mL [12, 55]). Nevertheless, given the level of protection seen here in the presence of supraphysiologic IL-1 insults level, we anticipate that in the presence of lower, more clinically relevant levels, protection would similarly be afforded. As described in these studies, we reason that in free swelling conditions, in the absence of any mechanical loading, a higher concentration of cytokine would be necessary to elicit an injurious effect relatively quickly. In fact, previous studies have demonstrated that engineered cartilage is insensitive to interleukin at ≤ 0.01 ng/mL, and exhibits an all-or-nothing response at IL concentrations from 0.1 to 10 ng/mL [11]. Theoretical modeling of dynamic deformational loading of hydrogels such as agarose have predicted that loading leads to enhanced transport of soluble factors such as growth factors and cytokines [56–59]. Accordingly, it may be possible to utilize more physiologic levels of cytokines in tandem with dynamic loading protocols in future evaluations of preconditioning schemes for tissue-engineered cartilage [26] to gain further insights into clinical feasibility.

## Conclusions

Here, we have described a novel method for desensitizing engineered cartilage to cytokine exposure, effectively protecting constructs from complete catabolic degradation. Though preconditioning protocols have been investigated and are widely recognized for their use in hypoxic and ischemic conditions, this is the first time such a protocol has been applied for protection against cytokine insult. Preconditioning with a low dose of IL-1α immediately is capable of protecting the construct against subsequent catabolic destruction from exposure to injurious levels of cytokine and provides an intriguing potential for using this technique for clinical therapeutic applicability.

## Abbreviations

ANOVA: Analysis of variance
CoCl_2_: Cobalt chloride
DMEM: Dulbecco’s modified Eagle’s medium
ECM: Extracellular matrix
E_Y_: Young’s modulus
FGF: Fibroblast growth factor
G*: Dynamic modulus
GAG: Glycosaminoglycan
HIF: Hypoxia-inducible factor
IACUC: International Animal Care and Use Committee
IL-1: Interleukin-1
MAP: Mitogen-activated protein
NGS: normal goat serum
OA: Osteoarthritis
OHP: Orthohydroxyproline
PDGF: Platelet-derived growth factor
TGF: Transforming growth factor
TNF: Tumor necrosis factor

## References

[1] Mow VC, Bachrach NM, Setton LA, Guilak F, Mow VC, Guilak F, Tran-Son-Tay R, Hochmuth RM (1994). Stress, strain, pressure, and flow fields in articular cartilage and chondrocytes. Cell Mechanics and Cellular Engineering.

[2] Tew S, Redman S, Kwan A, Walker E, Khan I, Dowthwaite G (2001). Differences in repair responses between immature and mature cartilage. Clin Orthop Relat Res.

[3] Goldring MB, Otero M, Plumb DA, Dragomir C, Favero M, El Hachem K (2011). Roles of inflammatory and anabolic cytokines in cartilage metabolism: signals and multiple effectors converge upon MMP-13 regulation in osteoarthritis. Eur Cell Mater.

[4] Lee JH, Fitzgerald JB, DiMicco MA, Grodzinsky AJ (2005). Mechanical injury of cartilage explants causes specific time-dependent changes in chondrocyte gene expression. Arthritis Rheum.

[5] Stevens AL, Wishnok JS, White FM, Grodzinsky AJ, Tannenbaum SR (2009). Mechanical injury and cytokines cause loss of cartilage integrity and upregulate proteins associated with catabolism, immunity, inflammation, and repair. Mol Cell Proteomics.

[6] Fan J, Varshney RR, Ren L, Cai D, Wang D-A (2009). Synovium-derived mesenchymal stem cells: a new cell source for musculoskeletal regeneration. Tissue Eng Part B Rev.

[7] Pelletier JP, Roughley PJ, DiBattista J, McCollum R, Martel-Pelletier J (1991). Are cytokines involved in osteoarthritic pathophysiology?. Semin Arthritis Rheum.

[8] Sellam J, Berenbaum F (2010). The role of synovitis in pathophysiology and clinical symptoms of osteoarthritis. Nat Rev Rheumatol.

[9] van den Berg WB, Joosten LA, van de Loo FA (1999). TNF alpha and IL-1 beta are separate targets in chronic arthritis. Clin Exp Rheumatol.

[10] Lima EG, Tan AR, Tai T, Bian L, Stoker AM, Ateshian GA (2008). Differences in interleukin-1 response between engineered and native cartilage. Tissue Eng Part A.

[11] Lima EG, Tan AR, Tai T, Bian L, Ateshian GA, Cook JL (2008). Physiologic deformational loading does not counteract the catabolic effects of interleukin-1 in long-term culture of chondrocyte-seeded agarose constructs. J Biomech.

[12] Wilusz RE, Weinberg JB, Guilak F, McNulty AL (2008). Inhibition of integrative repair of the meniscus following acute exposure to interleukin-1 in vitro. J Orthop Res.

[13] Aydelotte MB, Raiss RX, Caterson B, Kuettner K (1992). Influence of interleukin-1 on the morphology and proteoglycan metabolism of cultured bovine articular chondrocytes. Connect Tissue Res.

[14] Temple MM, Xue Y, Chen MQ, Sah RL (2006). Interleukin-1alpha induction of tensile weakening associated with collagen degradation in bovine articular cartilage. Arthritis Rheum.

[15] Murata M, Bonassar LJ, Wright M, Mankin HJ, Towle CA (2003). A role for the interleukin-1 receptor in the pathway linking static mechanical compression to decreased proteoglycan synthesis in surface articular cartilage. Arch Biochem Biophys.

[16] Lotz M (2001). Cytokines in cartilage injury and repair. Clin Orthop Relat Res.

[17] Smeets TJM, Barg EC, Kraan MC, Smith MD, Breedveld FC, Tak PP (2003). Analysis of the cell infiltrate and expression of proinflammatory cytokines and matrix metalloproteinases in arthroscopic synovial biopsies: comparison with synovial samples from patients with end stage, destructive rheumatoid arthritis. Ann Rheum Dis.

[18] Rotter N, Ung F, Roy AK, Vacanti M, Eavey RD, Vacanti CA (2005). Role for interleukin 1alpha in the inhibition of chondrogenesis in autologous implants using polyglycolic acid-polylactic acid scaffolds. Tissue Eng.

[19] Haider HK, Ashraf M (2010). Preconditioning and stem cell survival. J Cardiovasc Transl Res.

[20] Kamota T, Li TS, Morikage N, Murakami M, Ohshima M, Kubo M (2009). Ischemic pre-conditioning enhances the mobilization and recruitment of bone marrow stem cells to protect against ischemia/reperfusion injury in the late phase. J Am Coll Cardiol.

[21] Kubo M, Li TS, Suzuki R, Shirasawa B, Morikage N, Ohshima M (2008). Hypoxic preconditioning increases survival and angiogenic potency of peripheral blood mononuclear cells via oxidative stress resistance. Am J Physiol Heart Circ Physiol.

[22] Pasha Z, Wang Y, Sheikh R, Zhang D, Zhao T, Ashraf M (2008). Preconditioning enhances cell survival and differentiation of stem cells during transplantation in infarcted myocardium. Cardiovasc Res.

[23] Murry CE, Jennings RB, Reimer KA (1986). Preconditioning with ischemia: a delay of lethal cell injury in ischemic myocardium. Circulation.

[24] Tyler JA (1989). Insulin-like growth factor 1 can decrease degradation and promote synthesis of proteoglycan in cartilage exposed to cytokines. Biochem J.

[25] Hung CT, Mauck RL, Wang CCB, Lima EG, Ateshian GA (2004). A paradigm for functional tissue engineering of articular cartilage via applied physiologic deformational loading. Ann Biomed Eng.

[26] Lima EG, Bian L, Ng KW, Mauck RL, Byers BA, Tuan RS (2007). The beneficial effect of delayed compressive loading on tissue-engineered cartilage constructs cultured with TGF-beta3. Osteoarthr Cartil.

[27] Ng KW, Lima EG, Bian L, O'Conor CJ, Jayabalan PS, Stoker AM (2010). Passaged adult chondrocytes can form engineered cartilage with functional mechanical properties: a canine model. Tissue Eng Part A.

[28] Cook JL, Anderson CC, Kreeger JM, Tomlinson JL (2000). Effects of human recombinant interleukin-1beta on canine articular chondrocytes in three-dimensional culture. Am J Vet Res.

[29] Kuroki K, Stoker AM, Cook JL (2005). Effects of proinflammatory cytokines on canine articular chondrocytes in a three-dimensional culture. Am J Vet Res.

[30] Cook JL, Hung CT, Kuroki K, Stoker AM, Cook CR, Pfeiffer FM (2014). Animal models of cartilage repair. Bone Joint Res.

[31] Hu X, Yu SP, Fraser JL, Lu Z, Ogle ME, Wang J-A (2008). Transplantation of hypoxia-preconditioned mesenchymal stem cells improves infarcted heart function via enhanced survival of implanted cells and angiogenesis. J Thorac Cardiovasc Surg.

[32] Nanduri J, Yuan G, Kumar GK, Semenza GL, Prabhakar NR (2008). Transcriptional responses to intermittent hypoxia. Respir Physiol Neurobiol.

[33] Sampat SR, O'Connell GD, Fong JV, Alegre-Aguarón E, Ateshian GA, Hung CT (2011). Growth factor priming of synovium-derived stem cells for cartilage tissue engineering. Tissue Eng Part A.

[34] Soltz MA, Ateshian GA (1998). Experimental verification and theoretical prediction of cartilage interstitial fluid pressurization at an impermeable contact interface in confined compression. J Biomech.

[35] Kelly T-AN, Ng KW, Wang CC-B, Ateshian GA, Hung CT (2006). Spatial and temporal development of chondrocyte-seeded agarose constructs in free-swelling and dynamically loaded cultures. J Biomech.

[36] Stegeman H, Stalder K (1967). Determination of hydroxyproline. Clin Chim Acta.

[37] Bian L, Lima EG, Angione SL, Ng KW, Williams DY, Xu D (2008). Mechanical and biochemical characterization of cartilage explants in serum-free culture. J Biomech.

[38] Bian L, Fong JV, Lima EG, Stoker AM, Ateshian GA, Cook JL (2010). Dynamic mechanical loading enhances functional properties of tissue-engineered cartilage using mature canine chondrocytes. Tissue Eng Part A.

[39] Gilbert JE (1998). Current treatment options for the restoration of articular cartilage. Am J Knee Surg.

[40] Adkisson HD, Martin JA, Amendola RL, Milliman C, Mauch KA, Katwal AB (2010). The potential of human allogeneic juvenile chondrocytes for restoration of articular cartilage. Am J Sports Med.

[41] Patel HH, Gross ER, Peart JN, Hsu AK, Gross GJ (2005). Sarcolemmal KATP channel triggers delayed ischemic preconditioning in rats. Am J Physiol Heart Circ Physiol.

[42] Haider KH, Kim HW, Ashraf M (2009). Hypoxia-inducible factor-1alpha in stem cell preconditioning: mechanistic role of hypoxia-related micro-RNAs. J Thorac Cardiovasc Surg.

[43] Rosova I, Dao M, Capoccia B, Link D, Nolta JA (2008). Hypoxic preconditioning results in increased motility and improved therapeutic potential of human mesenchymal stem cells. Stem Cells.

[44] Tang YL, Zhu W, Cheng M, Chen L, Zhang J, Sun T (2009). Hypoxic preconditioning enhances the benefit of cardiac progenitor cell therapy for treatment of myocardial infarction by inducing CXCR4 expression. Circ Res.

[45] Sartori-Cintra AR, de Mara CS, Argolo DL, Coimbra IB (2012). Regulation of hypoxia-inducible factor-1α (HIF-1α) expression by interleukin-1β (IL-1 β), insulin-like growth factors I (IGF-I) and II (IGF-II) in human osteoarthritic chondrocytes. Clinics (Sao Paulo).

[46] Pfander D, Gelse K (2007). Hypoxia and osteoarthritis: how chondrocytes survive hypoxic environments. Curr Opin Rheumatol.

[47] Emerling BM, Platanias LC, Black E, Nebreda AR, Davis RJ, Chandel NS (2005). Mitochondrial reactive oxygen species activation of p38 mitogen-activated protein kinase is required for hypoxia signaling. Mol Cell Biol.

[48] Ashwell JD (2006). The many paths to p38 mitogen-activated protein kinase activation in the immune system. Nat Rev Immunol.

[49] Quinn TM, Schmid P, Hunziker EB, Grodzinsky AJ (2002). Proteoglycan deposition around chondrocytes in agarose culture: construction of a physical and biological interface for mechanotransduction in cartilage. Biorheology.

[50] Kelly T-AN, Wang CCB, Mauck RL, Ateshian GA, Hung CT (2004). Role of cell-associated matrix in the development of free-swelling and dynamically loaded chondrocyte-seeded agarose gels. Biorheology.

[51] Tan AR, Dong EY, Ateshian GA, Hung CT (2010). Response of engineered cartilage to mechanical insult depends on construct maturity. Osteoarthr Cartil.

[52] van Osch GJ, van den Berg WB, Hunziker EB, Hauselmann HJ (1998). Differential effects of IGF-1 and TGF beta-2 on the assembly of proteoglycans in pericellular and territorial matrix by cultured bovine articular chondrocytes. Osteoarthr Cartil.

[53] Xu C, Oyajobi BO, Frazer A, Kozaci LD, Russell RG, Hollander AP (1996). Effects of growth factors and interleukin-1 alpha on proteoglycan and type II collagen turnover in bovine nasal and articular chondrocyte pellet cultures. Endocrinology.

[54] Li KW, Wang AS, Sah RL (2003). Microenvironment regulation of extracellular signal-regulated kinase activity in chondrocytes: effects of culture configuration, interleukin-1, and compressive stress. Arthritis Rheum.

[55] McNulty AL, Rothfusz NE, Leddy HA, Guilak F (2013). Synovial fluid concentrations and relative potency of interleukin-1 alpha and beta in cartilage and meniscus degradation. J Orthop Res.

[56] Mauck RL, Hung CT, Ateshian GA (2003). Modeling of neutral solute transport in a dynamically loaded porous permeable gel: implications for articular cartilage biosynthesis and tissue engineering. J Biomech Eng.

[57] Mauck RL, Nicoll SB, Seyhan SL, Ateshian GA, Hung CT (2003). Synergistic action of growth factors and dynamic loading for articular cartilage tissue engineering. Tissue Eng.

[58] Albro MB, Chahine NO, Li R, Yeager K, Hung CT, Ateshian GA (2008). Dynamic loading of deformable porous media can induce active solute transport. J Biomech.

[59] Chahine NO, Albro MB, Lima EG, Wei VI, DuBois CR, Hung CT (2009). Effect of dynamic loading on the transport of solutes into agarose hydrogels. Biophys J.

